# Heterogeneity in national U.S. mortality trends within heart disease subgroups, 2000–2015

**DOI:** 10.1186/s12872-017-0630-2

**Published:** 2017-07-18

**Authors:** Stephen Sidney, Charles P. Quesenberry, Marc G. Jaffe, Michael Sorel, Alan S. Go, Jamal S. Rana

**Affiliations:** 1 Division of Research, Kaiser Permanente Northern California, 2000 Broadway, Oakland, CA 94612 USA; 2 Department of Endocrinology, Kaiser Permanente South San Francisco Medical Center, South San Francisco, CA USA; 3 Departments of Epidemiology, Biostatistics and Medicine, University of California, San Francisco, San Francisco, CA USA; 4 Department of Health Research and Policy, Stanford University School of Medicine, Stanford, CA USA; 5 Department of Cardiology, Kaiser Permanente Oakland Medical Center, Oakland, CA USA; 6 Department of Medicine, University of California, San Francisco, San Francisco, CA USA

**Keywords:** Mortality rate, Heart disease, Coronary heart disease, Heart failure, Epidemiology

## Abstract

**Background:**

The long-term downward national U.S. trend in heart disease-related mortality slowed substantially during 2011–2014 before turning upward in 2015. Examining mortality trends in the major subgroups of heart disease may provide insight into potentially more targeted and effective prevention and treatment approaches to promote favorable trajectories. We examined national trends between 2000 and 2015 in mortality attributed to major heart disease subgroups including ischemic heart disease, heart failure, and all other types of heart disease.

**Methods:**

Using the Centers for Disease Control and Prevention Wide-ranging Online Data for Epidemiologic Research (WONDER) data system, we determined national trends in age-standardized mortality rates attributed to ischemic heart disease, heart failure, and other heart diseases from January 1, 2000, to December 31, 2011, and from January 1, 2011, to December 31, 2015. Annual rate of changes in mortality attributed to ischemic heart disease, heart failure, and other heart diseases for 2000–2011 and 2011–2015 were compared.

**Results:**

Death attributed to ischemic heart disease declined from 2000 to 2015, but the rate of decline slowed from 4.96% (95% confidence interval 4.77%–5.15%) for 2000–2011 to 2.66% (2.00%–3.31%) for 2011–2015. In contrast, death attributed to heart failure and all other causes of heart disease declined from 2000 to 2011 at annual rates of 1.94% (1.77%–2.11%) and 0.64% (0.44%–0.82%) respectively, but increased from 2011 to 2015 at annual rates of 3.73% (3.21% 4.26%) and 1.89% (1.33–2.46%). Differences in 2000–2011 and 2011–2015 decline rates were statistically significant for all 3 endpoints overall, by sex, and all race/ethnicity groups except Asian/Pacific Islanders (heart failure only significant) and American Indian/Alaskan Natives.

**Conclusions:**

While the long-term decline in death attributed to heart disease slowed between 2011 and 2014 nationally before turning upward in 2015, heterogeneity existed in the trajectories attributed to heart disease subgroups, with ischemic heart disease mortality continuing to decline while death attributed to heart failure and other heart diseases switched from a downward to upward trend. While systematic efforts to prevent and treat ischemic heart disease continue to be effective, urgent attention is needed to address the challenge of heart failure.

## Background

We recently reported that the rate of decline of death attributed to total cardiovascular disease (CVD) and to heart disease (HD) in the U.S. had decelerated substantially between 2011 and 2014 [1], with the annualized percent decline in CVD and HD mortality decreasing from 3.79% and 3.69% respectively for 2000–2011 to 0.65% and 0.76% for 2011–2014. We suggested that HD mortality might increase in 2015 [1] which was confirmed by the recent report of a 0.9% increase from 167.0 to 168.5 per 100,000 person-years from 2014 to 2015, the first year-to-year increase since 1992–93 [2, 3].

HD-related death encompasses a wide range of heart conditions. Thus, from both prevention and intervention perspectives, it is important to further delineate trends in subcategories of HD-related death. We studied mortality trends in the two largest subgroups of HD (ischemic heart disease [IHD] and heart failure [HF]) and in all other HD combined.

## Methods

Mortality rates between 2000 and 2015 were ascertained using the U.S. Centers for Disease Control and Prevention’s Wide-Ranging Online Data for Epidemiologic Research (CDC WONDER) dataset, which includes the assigned cause of death from all death certificates filed in the 50 states and the District of Columbia [3]. Categorization of the presumed cause of death used *International Statistical Classification of Diseases and Related Health Problems, Tenth Edition* codes as follows: HD (codes I00-I09, I11, I13, and I20-I51), IHD (I20-I25), HF (I50), and all other causes of HD (I00-I09, I11, I13, I26-I49, and I51).

This study did not require institutional review board approval because it analyzes government-issued public use data without individual identifiable information.

Age-standardized mortality rates (AAMR) were calculated using the direct method, with the 2000 U.S. Census as the standard population using the following age categorization: younger than 1 year, 1 to 4, 5 to 14, 15 to 24, 25 to 34, 35 to 44, 45 to 54, 55 to 64, 65 to 74, 75 to 84, and 85 years or older [4]. Poisson regression with allowance for overdispersion was used for point and interval estimation of age-adjusted annual rates of change for January 1, 2000, to December 31, 2011, and January 1, 2011, to December 31, 2015.

## Results

Mortality rates from 2000 to 2015 for HD and HD subgroups are shown in Table 1, with the largest subgroup being IHD. Compared to 2014, in 2015, an increase in overall HD occurred in men (0.4%), women (1.4%), and in all racial-ethnic groups except NH Blacks in which HD mortality decreased by 0.3%. The 2015 mortality rate for each HD subgroup was higher in men than in women. By race-ethnicity, NH blacks had the highest mortality rate for each HD subcategory, followed by NH whites, NH American Indian/Alaskan Natives, Hispanics, and NH Asian/Pacific Islanders.

**Table 1:** Age-adjusted mortality rates for all heart disease, ischemic heart disease, heart failure, and all other CHD, United States, 2000–2015

		Heart disease		Ischemic HD		Heart failure		All other HD	
Year	N = Population	n = deaths	AAMR^a^	n = deaths	AAMR	n = deaths	AAMR	n = deaths	AAMR
2000	281,421,906	710,760	257.6	515,204	186.8	55,704	20.3	139,852	50.6
2001	284,968,955	700,142	249.5	502,189	179.0	56,934	20.4	141,019	50.2
2002	287,625,193	696,947	244.6	494,382	173.5	56,494	19.9	146,071	51.2
2003	290,107,933	685,089	236.3	480,028	165.6	57,448	19.9	147,613	50.9
2004	292,805,298	652,486	221.6	451,326	153.2	57,120	19.5	144,040	48.9
2005	295,516,599	652,091	216.8	445,687	148.2	58,933	19.7	147,471	49.0
2006	298,379,912	631,636	205.5	425,425	138.3	60,337	19.7	145,874	47.5
2007	301,231,207	616,067	196.1	406,351	129.2	56,565	18.0	153,151	48.8
2008	304,093,966	616,828	192.1	405,309	126.1	56,830	17.7	154,689	48.3
2009	306,771,529	599,413	182.8	386,324	117.7	56,410	17.2	156,679	47.9
2010	308,745,538	597,689	179.1	379,559	113.6	57,757	17.3	160,373	48.2
2011	311,591,917	596,577	173.7	375,295	109.2	58,309	16.9	162,973	47.7
2012	313,914,040	599,711	170.5	371,469	105.4	60,341	17.1	167,901	48.0
2013	316,128,839	611,105	169.8	370,213	102.6	65,120	18.0	175,772	49.1
2014	318,857,056	614,348	167.0	364,593	98.8	68,626	18.6	181,129	49.6
2015	321,418,820	633,842	168.5	366,801	97.2	75,251	19.9	191,790	51.4

*Abbreviations*: *HD* heart disease, *AAMR* age-adjusted mortality rate

^a^Age-adjusted mortality rate per 100,000 person-years, directly standardized to the 2000 U.S. population

**Fig. 1:**
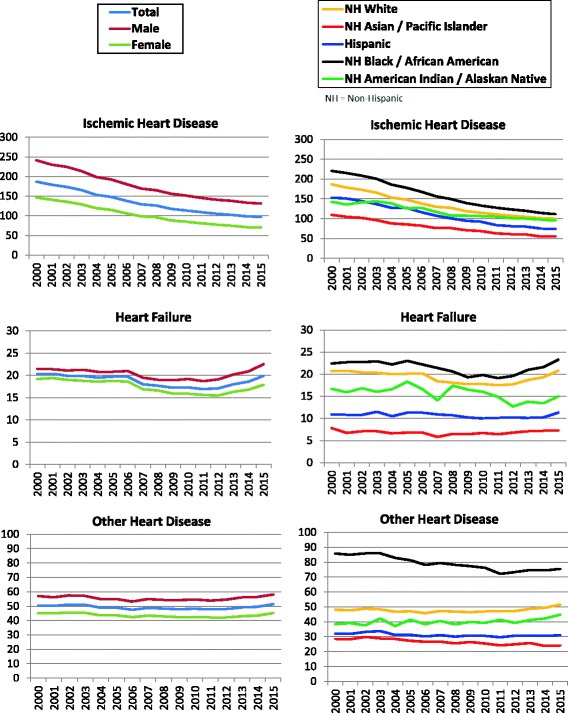
Age-adjusted mortality in U.S., 2000–2015 by sex and race-ethnicity. Legend: Total, Male, Female, NH White, NH Asian/Pacific Islander, Hispanic, NH Black, NH American Indian/Alaskan Native (NH – Non-Hispanic)

The rate of decline in death attributed to IHD slowed in 2011–2015, with mean annual rate of change of −2.66% compared to −4.96% for 2000–2011 (Table 2 **,** Fig. 1). The difference in the rate of change between the two time periods was statistically significant overall, in each sex and, among NH whites, NH blacks, and Hispanics.

**Table 2:** Age-adjusted mortality rates and annual rates of change for ischemic heart disease, heart failure, and other heart disease for time periods 2000–2011 and 2011–2015, United States

	AAMR			Annual rate of change (%)^a^		
Year(s)	2000	2011	2015	2000–2011	2011–2015	*p*-value^b^
Ischemic heart disease						
Total	186.8	109.2	97.2	−4.96 (−5.15 to −4.77)	−2.66 (−3.31 to −2.00)	<0.001
Total male	241.4	145.6	131.2	−4.63 (−4.82 to −4.44)	−2.10 (−2.75 to −1.45)	<0.001
Total female	146.5	81.0	70.5	−5.49 (−5.69 to −5.29)	−3.69 (−4.29 to −2.88)	<0.001
NH White	186.6	111.1	99.7	−4.85 (−5.05 to −4.64)	−2.34 (−3.05 to −1.63)	<0.001
NH Asian/PI	109.7	63,2	55.1	−4.71 (−5.03 to −4.43)	−3.75 (−4.64 to −2.85)	0.08
Hispanic	153.2	84.2	74.5	−5.38 (−5.64 to −5.12)	−3.39 (−4.21 to −2.58)	<0.001
NH Black	220.4	127.9	111.3	−5.06 (−5.26 to −4.86)	−3.16 (−3.93 to −2.49)	0.003
NH AI/AN	142.7	104.8	95.2	−3.04 (−3.55 to −2.52)	−1.23 (−2.79 to 0.36)	0.06
Heart failure						
Total	20.3	16.9	19.9	−1.94 (−2.11 to −1.77)	3.73 (3.21 to 4.26)	<0.001
Total male	21.5	18.7	22.5	−1.51 (−1.70 to −1.31)	4.58 (4.00 to 5.17)	<0.001
Total female	19.2	15.6	17.9	−2.26 (−2.44 to −2.09)	2.99 (2.43 to 3.56)	<0.001
NH White	20.7	17.5	20.8	−1.86 (−2.02 to −1.70)	4.10 (3.60 to 4.61)	<0.001
NH Asian/PI	7.8	6.4	7.3	−0.95 (−1.68 to −0.22)	4.14 (2.28 to 6.04)	<0.001
Hispanic	10.9	10.7	11.3	−0.94 (−1.40 to −0.48)	1.87 (0.65 to 3.11)	<0.001
NH Black	22.4	19.1	23.3	−1.66 (−1.98 to −1.34)	4.40 (3.43 to 5.37)	<0.001
NH AI/AN	16.7	14.9	15.0	−1.12 (−2.14 to −0.09)	−2.08 (−4.95 to 0.89)	0.60
Other heart disease						
Total	50.6	47.7	51.4	−0.63 (−0.82 to −0.44)	1.89 (1.33 to 2.46)	<0.001
Total male	57.1	53.9	58.1	−0.59 (−0.79 to 0.39)	2.04 (1.45 to 2.63)	<0.001
Total female	45.1	42.1	45.2	−0.69 (−0.90 to −0.49)	1.73 (1.11 to 2.36)	<0.001
NH White	48.1	47.1	51.5	−0.34 (−0.55 to −0.22)	2.52 (1.87 to 3.17)	<0.001
NH Asian/PI	28.6	24.2	24.2	−1.70 (−2.06 to −1.33)	−0.38 (−1.37 to 0.61)	0.04
Hispanic	31.9	31.4	31.9	−0.93 (−1.19 to −0.66)	0.78 (−0.06 to 1.51)	<0.001
NH Black	85.7	72.1	75.5	−1.51 (−1.67 to −1.35)	0.42 (−0.06 to 0.91)	<0.001
NH AI/AN	38.5	41.3	44.8	0.55 (−0.24 to 1.34)	2.57 (0.41 to 4.78)	0.14

*Abbreviations*: *AAMR* age-adjusted mortality rate, *NH* non-Hispanic, *PI* Pacific Islander, *AI*/*AN* American Indian/Alaskan Native

^a^Annual rate of change age-adjusted by Poisson regression

^b^ *p*-value for difference in annual rate of change between 2000 and 2011 and 2011–2015 time periods

In sharp contrast, mortality rates attributed to HF and all other HD declined from 2000 to 2011, but then increased from 2011 to 2015 (Table 2, Fig. 1). These patterns were evident in both sexes (Tables 3 and 4 and Fig. 1) and in all race-ethnicity groups except NH American Indian/Alaskan Natives (Tables 5, 6, 7, 8 and 9 **,** Fig. 1). From 2011 to 2015, the mean annual rate of increase was 3.73% for HF-related mortality and 1.89% for all other HD mortality in the total population. The difference in the rate of change between the two time periods was statistically significant overall in each sex, and in all race-ethnicity groups except NH American Indian/Alaskan Natives for HF and other HD mortality as well as NH Asian/Pacific Islander for other HD mortality (Table 2 **)**. Trends in crude mortality rates (Table 10) for HD and each HD subgroup were similar to age-standardized mortality trends.

**Table 3:** Males (age-adjusted)

Trends in mortality in United States from 2000 to 2015 by gender and race-ethnicity									
		Heart disease		Ischemic HD		Heart failure		All other HD	
Year	(n = Population)	(n = deaths)	AAMR	(n = deaths)	AAMR	(n = deaths)	AAMR	(n = deaths)	AAMR
2000	138,053,563	344,807	320.0	260,574	241.4	21,175	21.5	63,058	57.1
2001	139,891,492	339,095	307.8	254,005	230.2	21,632	21.4	63,458	56.1
2002	141,230,559	340,933	303.4	252,760	224.7	21,698	21.1	66,475	57.6
2003	142,428,897	336,095	292.3	246,342	213.9	22,427	21.3	67,326	57.1
2004	143,828,012	321,973	274.1	233,538	198.4	22,292	20.8	66,143	54.9
2005	145,197,078	322,841	268.2	232,115	192.3	23,026	20.8	67,700	55.0
2006	146,647,265	315,706	254.9	224,510	180.7	23,918	21.0	67,278	53.2
2007	148,064,854	309,821	243.7	216,050	169.2	22,914	19.5	70,857	55.0
2008	149,489,951	311,201	238.5	216,248	165.1	23,017	19.0	71,936	54.3
2009	150,807,454	307,225	229.4	210,069	156.2	23,563	18.9	73,593	54.2
2010	151,781,326	307,384	225.1	207,580	151.3	24,385	19.2	75,419	54.6
2011	153,290,819	308,398	218.1	206,908	145.6	24,609	18.7	76,881	53.9
2012	154,492,067	312,491	214.7	206,685	141.1	26,036	19.1	79,770	54.5
2013	155,651,602	321,347	214.5	208,515	138.2	28,513	20.2	84,319	56.1
2014	156,936,487	325,077	210.9	207,412	133.5	30,339	20.9	87,326	56.5
2015	158,229,297	335,002	211.8	209,298	131.2	33,667	22.5	92,037	58.1

Age-adjusted mortalilty rate per 100,000 person-years, directly standardized to the 2000 U.S. population

**Table 4:** Female (age-adjusted)

Trends in mortality in United States from 2000 to 2015 by gender and race-ethnicity									
		Heart disease		Ischemic HD		Heart failure		All other HD	
Year	(n = Population)	(n = deaths)	AAMR	(n = deaths)	AAMR	(n = deaths)	AAMR	(n = deaths)	AAMR
2000	143,368,343	365,953	210.9	254,630	146.5	34,529	19.2	76,794	45.1
2001	145,077,463	361,047	205.4	248,184	140.9	35,302	19.4	77,561	45.0
2002	146,394,634	356,014	200.3	241,622	135.7	34,796	19.0	79,596	45.6
2003	147,679,036	348,994	193.7	233,686	129.4	35,021	18.8	80,287	45.4
2004	148,977,286	330,513	181.5	217,788	119.4	34,828	18.6	77,897	43.5
2005	150,319,521	329,250	177.5	213,572	115.0	35,907	18.8	79,771	43.7
2006	151,732,647	315,930	167.2	200,915	106.3	36,419	18.6	78,596	42.3
2007	153,166,353	306,246	159.0	190,301	98.8	33,651	16.9	82,294	43.4
2008	154,604,015	305,627	155.9	189,061	96.3	33,813	16.6	82,753	42.9
2009	155,964,075	292,188	146.6	176,255	88.4	32,847	15.9	83,086	42.3
2010	156,964,212	290,305	143.3	171,979	84.9	33,372	15.9	84,954	42.5
2011	158,301,098	288,179	138.7	168,387	81.0	33,700	15.6	86,092	42.1
2012	159,421,973	287,220	135.5	164,784	77.8	34,305	15.5	88,131	42.2
2013	160,477,237	289,758	134.3	161,698	74.9	36,607	16.3	91,453	43.0
2014	161,920,569	289,271	131.8	157,181	71.6	38,287	16.8	93,803	43.4
2015	163,189,523	298,840	133.6	157,503	70.5	41,584	17.9	99,753	45.2

Age-adjusted mortalilty rate per 100,000 person-years, directly standardized to the 2000 U.S. population

**Table 5:** Non-Hispanic White (age-adjusted)

Trends in mortality in United States from 2000 to 2015 by gender and race-ethnicity									
		Heart disease		Ischemic HD		Heart failure		All other HD	
Year	(n = Population)	(n = deaths)	AAMR	(n = deaths)	AAMR	(n = deaths)	AAMR	(n = deaths)	AAMR
2000	197,324,684	594,465	255.5	434,505	186.6	48,782	20.7	111,178	48.1
2001	197,842,671	582,349	247.2	420,959	178.5	49,788	20.8	111,602	47.7
2002	198,101,982	577,761	242.5	413,230	173.2	49,162	20.4	115,369	48.8
2003	198,289,486	565,808	234.2	400,101	165.5	49,788	20.3	115,919	48.4
2004	198,619,903	537,512	220.1	374,900	153.3	49,628	20	112,984	46.7
2005	198,880,984	535,101	215.5	368,505	148.3	50,835	20.1	115,761	47.1
2006	199,200,396	516,883	204.5	350,356	138.6	52,125	20.2	114,402	45.8
2007	199,492,421	502,683	195.5	334,047	129.9	48,480	18.4	120,156	47.2
2008	199,783,797	503,096	192.4	333,378	127.4	48,518	18.1	121,200	46.8
2009	199,993,079	485,779	182.9	315,810	118.9	48,156	17.7	121,813	46.4
2010	200,127,372	483,973	179.9	309,492	115.0	49,253	17.8	125,228	47.0
2011	200,423,243	482,979	175.6	305,486	111.1	49,605	17.5	127,888	47.1
2012	200,698,847	481,991	172.3	300,439	107.4	50,922	17.7	130,630	47.2
2013	200,918,513	488,817	171.8	297,501	104.6	54,787	18.7	136,529	48.6
2014	201,048,793	489,926	169.9	291,879	101.2	57,522	19.3	140,525	49.4
2015	201,242,281	503,172	171.9	291,850	99.7	62,649	20.8	148,673	51.5

Age-adjusted mortalilty rate per 100,000 person-years, directly standardized to the 2000 U.S. population

**Table 6:** Non-Hispanic Asian/Pacific Islander (age-adjusted)

Trends in mortality in United States from 2000 to 2015 by gender and race-ethnicity									
		Heart disease		Ischemic HD		Heart failure		All other HD	
Year	(n = Population)	(n = deaths)	AAMR	(n = deaths)	AAMR	(n = deaths)	AAMR	(n = deaths)	AAMR
2000	11,355,553	8949	146.1	6689	109.7	418	7.8	1842	28.6
2001	11,983,178	9291	139.5	6916	104.5	392	6.7	1983	28.3
2002	12,472,384	9814	139.2	7159	102.3	445	7.1	2210	29.9
2003	12,942,337	9934	132.5	7221	96.5	474	7.1	2239	28.9
2004	13,406,530	9756	123.4	6954	88.2	475	6.6	2327	28.5
2005	13,888,295	10,281	119.8	7329	85.7	519	6.8	2433	27.3
2006	14,375,996	10,457	115.7	7430	82.3	556	6.8	2471	26.6
2007	14,854,701	10,394	108.6	7292	76.1	504	5.8	2598	26.7
2008	15,336,181	10,951	108.1	7705	76.1	606	6.5	2640	25.5
2009	15,793,995	11,134	103.8	7616	70.9	638	6.4	2880	26.5
2010	16,133,872	11,254	101.1	7683	69	694	6.7	2877	25.4
2011	16,579,709	11,406	93.8	7712	63.2	714	6.4	2980	24.2
2012	17,175,596	12,068	92.7	7959	61	825	6.8	3284	24.9
2013	17,693,870	13,064	93.2	8477	60.3	954	7.1	3633	25.7
2014	18,436,908	13,021	86.4	8360	55.3	1029	7.2	3632	23.8
2015	19,116,557	13,974	86.6	8921	55.1	1124	7.3	3929	24.2

Age-adjusted mortalilty rate per 100,000 person-years, directly standardized to the 2000 U.S. population

**Table 7:** Hispanic (age-adjusted)

Trends in mortality in United States from 2000 to 2015 by gender and race-ethnicity									
		Heart disease		Ischemic HD		Heart failure		All other HD	
Year	(n = Population)	(n = deaths)	AAMR	(n = deaths)	AAMR	(n = deaths)	AAMR	(n = deaths)	AAMR
2000	35,305,818	25,819	196	19,744	153.2	1270	10.9	4805	31.9
2001	37,144,096	27,090	193.7	20,664	151.1	1364	10.8	5062	31.9
2002	38,617,620	27,887	188.8	20,941	144.7	1412	10.8	5534	33.2
2003	40,049,429	28,298	182.1	20,783	136.8	1606	11.5	5909	33.8
2004	41,501,375	27,788	169.1	20,482	127.4	1545	10.5	5761	31.2
2005	43,023,614	29,555	170.4	21,774	127.9	1721	11.3	6060	31.3
2006	44,606,305	28,921	157.8	20,939	116.4	1830	11.3	6152	30.1
2007	46,196,853	29,021	149.5	20,452	107.5	1890	10.9	6679	31.1
2008	47,793,785	28,951	141.4	20,261	100.8	1966	10.7	6724	30.0
2009	49,327,489	29,611	135.8	20,228	94.7	2013	10.2	7370	30.9
2010	50,477,594	30,006	132.8	20,494	92.3	2024	10	7488	30.6
2011	52,045,277	30,385	123.9	20,326	84.2	2233	10.1	7826	29.6
2012	53,027,708	31,595	122	20,751	81.1	2404	10.2	8440	30.7
2013	54,071,370	33,243	121.2	21,788	80.3	2544	10.1	8911	30.7
2014	55,387,539	34,021	116	21,871	75.3	2742	10.2	9408	30.5
2015	56,592,793	36,401	116.9	23,055	74.5	3239	11.3	10,107	31.9

Age-adjusted mortalilty rate per 100,000 person-years, directly standardized to the 2000 U.S. population

**Table 8:** Non-Hispanic Black (age-adjusted)

Trends in mortality in United States from 2000 to 2015 by gender and race-ethnicity									
		Heart disease		Ischemic HD		Heart failure		All other HD	
Year	(n = Population)	(n = deaths)	AAMR	(n = deaths)	AAMR	(n = deaths)	AAMR	(n = deaths)	AAMR
2000	35,091,809	76,706	328.4	50,659	220.4	4936	22.4	21,111	85.7
2001	35,638,389	76,794	322.6	50,295	215.0	5094	22.7	21,405	84.9
2002	36,049,904	76,694	317.1	49,522	208.5	5143	22.7	22,029	85.9
2003	36,422,205	76,452	309.6	48,617	200.8	5294	22.9	22,541	85.9
2004	36,848,991	73,373	290.9	46,064	186.0	5198	22.2	22,111	82.8
2005	37,270,736	73,302	282.4	45,435	178.1	5570	23	22,297	81.3
2006	37,719,495	71,461	268.2	43,992	168.0	5524	22.2	21,945	78.1
2007	38,184,699	70,443	257.4	42,152	156.5	5464	21.4	22,827	79.4
2008	38,651,733	69,918	248.1	41,373	149.4	5415	20.6	23,130	78.2
2009	39,104,815	68,811	236.4	39,956	139.8	5290	19.3	23,565	77.3
2010	39,437,133	68,215	229.5	39,047	133.4	5497	19.8	23,671	76.2
2011	39,944,896	67,595	219.3	38,928	127.9	5492	19.1	23,175	72.1
2012	40,391,388	69,147	216.3	39,005	123.4	5879	19.6	24,263	73.3
2013	40,802,086	71,102	215.5	39,199	119.9	6518	21	25,385	74.7
2014	41,316,519	71,894	210.8	38,843	114.8	6962	21.6	26,089	74.4
2015	41,777,483	74,093	210.1	39,054	111.3	7772	23.3	27,267	75.5

Age-adjusted mortalilty rate per 100,000 person-years, directly standardized to the 2000 U.S. population

**Table 9:** Non-Hispanic American Indian/Alaskan Native (age-adjusted)

Trends in mortality in United States from 2000 to 2015 by gender and race-ethnicity									
		Heart disease		Ischemic HD		Heart failure		All other HD	
Year	(n = Population)	(n = deaths)	AAMR	(n = deaths)	AAMR	(n = deaths)	AAMR	(n = deaths)	AAMR
2000	2,344,042	2350	197.8	1688	142.7	171	16.7	491	38.5
2001	2,360,621	2353	190.6	1672	135.6	163	15.9	518	39.1
2002	2,383,303	2421	195.7	1744	141.3	182	16.8	495	37.6
2003	2,404,476	2634	201.6	1855	143.5	176	16.0	603	42.2
2004	2,428,499	2524	192.8	1795	138.9	187	16.6	542	37.2
2005	2,452,970	2576	185.7	1738	126.0	216	18.3	622	41.4
2006	2,477,720	2630	182.7	1810	127.6	208	16.6	612	38.4
2007	2,502,533	2557	171.6	1719	117.0	180	14.1	658	40.6
2008	2,528,470	2549	163.6	1671	108.0	230	17.4	648	38.2
2009	2,552,151	2654	164.2	1737	107.8	230	16.5	687	39.9
2010	2,569,567	2656	161.6	1747	106.3	217	16.0	692	39.3
2011	2,598,792	2805	161	1836	104.8	222	14.9	747	41.3
2012	2,620,501	2823	153.7	1878	101.7	201	12.7	744	39.3
2013	2,643,000	3002	155.5	1949	100.4	230	13.8	823	41.2
2014	2,667,297	3118	153.3	2009	97.8	233	13.4	876	42.2
2015	2,689,706	3303	154.9	2044	95.2	286	15.0	973	44.8

Age-adjusted mortalilty rate per 100,000 person-years, directly standardized to the 2000 U.S. population

**Table 10:** Crude mortality rate, total population rates (per 100,000 person years)

	Heart disease	Ischemic HD	Heart failure	All other HD
2000	252.6	183.1	19.8	49.7
2001	245.7	176.2	20.0	49.5
2002	242.3	171.9	19.6	50.8
2003	236.1	165.5	19.8	50.9
2004	222.8	154.1	19.5	49.2
2005	220.7	150.8	19.9	49.9
2006	211.7	142.6	20.2	48.9
2007	204.5	134.9	18.8	50.8
2008	202.8	133.3	18.7	50.9
2009	195.4	125.9	18.4	51.1
2010	193.6	122.9	18.7	51.9
2011	191.5	120.4	18.7	52.3
2012	191.0	118.3	19.2	53.5
2013	193.3	117.1	20.6	55.6
2014	192.7	114.3	21.5	56.8
2015	197.2	114.1	23.4	59.7

Five specific ICD-10 codes accounted for 63% of deaths attributed to other HD during 2011–2015. There was an increase in age-standardized mortality rates per 100,000 person-years from 9.7 to 11.1 for hypertensive HD (ICD-10 code I11), 5.2 to 6.3 (*p* < 0.001) for atrial fibrillation and flutter (ICD-10 code I48), and a decrease from 6.8 to 6.3 (*p* < 0.001) for cardiomyopathy (I42). Changes were not statistically significant for nonrheumatic aortic valve disorders (I35), 4.5 to 4.6 (*p* = 0.45); and cardiac arrest (I46), 4.4 to 4.3 (*p* = 0.48).

## Discussion

The increase in death attributed to HD in 2015 represents a notable landmark denoting a time where the impact of prevention efforts has been at least temporarily stalled. HD mortality increased across both sexes and most race-ethnicity groups. Although a slight decline was noted for NH blacks, HD-related death rates in this subgroup remain substantially higher than in other racial/ethnic groups.

While the continued decline in IHD mortality is encouraging, the rate of decline decreased by nearly 50% during the 2011–2015 period compared to 2000–2011. The decades-long epidemic of obesity and diabetes mellitus are likely important factors contributing the deceleration of the rate of decline of cardiovascular mortality nationally [1]. A recent study analyzing data from several cohort studies demonstrated a substantial decrease in the incidence of new-onset IHD between two time periods, with baseline exams conducted from 1983 to 1990 and 1996 to 2001, and showed that the fraction of CHD attributable to diabetes decreased over time [5]. However, the prevalence of diabetes has risen considerably from the time period that diabetes was assessed for these studies, [6] and populations now living with longer duration of diabetes have higher risk of CHD [7]. Additionally, follow-up ended in 2011, the year that the IHD mortality trend change occurred, so that the findings regarding the decreasing fraction of CHD attributable to diabetes are likely to not be as relevant to the current time period.

Several U.S.-based studies have shown decline in the incidence of acute myocardial infarction with follow-up through 2008–2011, [8–11] with one reporting additional follow-up showing continued decline through 2014 [12]. On the other hand, the prevalence of HF is on the rise [13]. The mortality trends for ischemic heart disease and HF since 2011 parallel these findings and are therefore plausible.

CVD remain a major cause of health loss internationally. Per the recent GBD (Global Burden of Disease) study, although dramatic declines in CVD occurred in regions with high socioeconomic status, only a gradual decrease or no change was noted in most other regions [14]. Of note, the data analyzed in our study used common groupings of ICD-10 codes to define heart disease and its subtypes such as IHD and all other HD in National Vital Statistics reports for the U.S. [15] that may be slightly different than codes used in GBD studies to define CVD and subtypes [16]. Therefore, the mortality numbers may vary. Similarly, in another study, trends in CHD and CVD mortality continue to be less favorable in Latin America than in Canada and in the U.S. [17].

The National Center for Health Statistics recently reported that deaths considered HF-related (i.e., HF reported anywhere on the death certification) declined from 2000 to 2012 but increased from 2012 to 2014 [18]. It is possible that HF is being inappropriately designated as the underlying cause of death in many instances [19]. This report noted that IHD was the underlying cause of death in 2014 for 23.9% of HF-related deaths in adults aged 45 years and older but did not report on the frequency of IHD as a listed cause of death when HF was recorded as the underlying cause of death. This might slightly attenuate the downward trend in the IHD mortality rate if HF is being designated as the underlying cause of death when it is due to IHD.

Another potential cause of misclassification of HF-related mortality is competing mortality with a non-CVD cause. While it is possible that declining cancer rates could result in the recent increasing trend in HF mortality and this year’s increase in HD mortality, it is unlikely since cancer mortality has been declining at a fairly stable rate of 1.5% per year since 2000 [1]. The most plausible sources for competing non-CVD mortality are diabetes (E10-E14) and chronic lower respiratory diseases (J40–47) which have declined minimally from 2011 to 2015 (data not shown).

It is well-recognized that HF is a major and growing public health problem. Earlier estimates from projection models for the U.S. suggest that the prevalence of HF will increase by 46% from 2012 to 2030 [13]. It has been suggested that the absence of a national surveillance system significantly impedes the ability to track and manage this expected increase in HF [20]. Given this, present CDC mortality data becomes an important indicator for burden of HF. Another matter of importance is a rising proportion of patients having HF with preserved ejection fraction (HFpEF), accounting for more than 50% of incident HF cases, and no definitive treatment to so far, has been proven effective in reducing the morbidity and mortality of HFpEF [21]. Further concomitant multiple comorbid conditions are frequent in this patient population, [22] with a recent analysis from Denmark showing an increasing prevalence of comorbidities, including diabetes mellitus and hypertension, especially in younger patients with HF [23]. It is plausible that the increasing prevalence of these comorbidities and lower death rates after acute myocardial infarction are contributing to increased HF-related mortality rates. Whereas better risk factor control strategies to prevent HF may reduce the incidence, [24] more effective treatments for patients with established HF would be expected to reduce case-fatality.

## Conclusions

While the mortality rate attributed to HD slowed substantially between 2011 and 2014 nationally before turning upward in 2015, trajectories among HD subgroups were heterogeneous, with IHD-related death continuing to decline while death attributed to HF and other causes of HD increased. While systematic efforts to prevent and treat IHD appear to be effective and require continued vigilance, an expanded focus on strategies to reduce deaths from HF and those attributed to other HD conditions appear needed. Finally, addressing the complex care of HF patients with multiple morbidities would likely need system-wide, multipronged health care interventions, with particularly urgent attention to developing more effective treatments for HFpEF [25].

## Abbreviations

AAMR: Age-adjusted mortality rate
CVD: Cardiovascular disease
HD: Heart disease
HF: Heart failure
ICD-10: International Statistic Classification of Diseases and Related Health Problems, Tenth Edition
IHD: Ischemic heart disease
